# SARS-CoV-2 Infection or COVID-19 mRNA Vaccination Elicits Partially Different Spike-Reactive Memory B Cell Responses in Naïve Individuals

**DOI:** 10.3390/vaccines13090944

**Published:** 2025-09-03

**Authors:** Lingling Yao, Noémi Becza, Georgia Stylianou, Magdalena Tary-Lehmann, Stephen M. Todryk, Greg A. Kirchenbaum, Paul V. Lehmann

**Affiliations:** 1 Research and Development, Cellular Technology Ltd. (CTL), Shaker Heights, OH 44122, USA; lingling.yao@immunospot.com (L.Y.); noemi.becza@immunospot.com (N.B.); greg.kirchenbaum@immunospot.com (G.A.K.); 2 Faculty of Health & Life Sciences, Northumbria University, Newcastle upon Tyne NE1 8ST, UK; georgia3.stylianou@northumbria.ac.uk (G.S.); stephen.todryk@northumbria.ac.uk (S.M.T.); 3 Contract Laboratory, Cellular Technology Ltd. (CTL), Shaker Heights, OH 44122, USA; magda.tary-lehmann@immunospot.com

**Keywords:** ELISPOT, FluoroSpot, immunological memory, humoral, Ig class, IgG subclass, plasma cell, immune monitoring

## Abstract

**Background:** The COVID-19 pandemic provided a unique opportunity to evaluate how the human immune system responded to a novel pathogen and to determine whether immune responses initiated through natural infection differ from those elicited by vaccination against the same antigen. Here, we provide a comprehensive analysis of SARS-CoV-2 Spike (S-antigen)-reactive memory B cells (B_mem_) elicited in previously immunologically naïve subjects following their first infection with the original Wuhan-Hu-1 (WH1)-like strain or their initial COVID-19 mRNA prime-boost regimen encoding the same WH1-S-antigen. In particular, we tested the hypothesis that the primary encounter of SARS-CoV-2 S-antigen in lung mucosal tissues during infection vs. intramuscular COVID-19 mRNA injection would elicit different B_mem_ responses. **Methods:** Cryopreserved peripheral blood mononuclear cell (PBMC) samples collected following primary infection with the WH1 strain or completion of the initial prime-boost vaccination regimen were tested in ImmunoSpot^®^ assays to assess the frequency, Ig class/subclass usage, and cross-reactivity of the S-antigen-reactive B_mem_ compartment; pre-pandemic blood draws served as naïve controls. **Results**: The B_mem_ repertoires generated post-infection vs. post-vaccination were found to be quite similar but with some subtle differences. In both cases, the prevalent induction of IgG1-expressing B_mem_ in similar frequencies was seen, ~30% of which targeted the receptor binding domain (RBD) of the WH1-S-antigen. Also, the extent of cross-reactivity with the future Omicron (BA.1) RBD was found to be similar for both cohorts. However, IgA^+^ B_mem_ were preferentially induced after infection, while IgG4^+^ B_mem_ were detected only after vaccination. **Conclusions:** B_mem_ elicited in naïve human subjects following SARS-CoV-2 infection or after WH1-S encoding mRNA vaccination were only subtly different, although the relevance of these differences as it relates to immune protection warrants further investigation. Our findings serve to illustrate the usefulness and feasibility of performing comprehensive monitoring of antigen-specific B cell memory in larger cohorts using the ImmunoSpot^®^ technique.

## 1. Introduction

Mucosal surfaces (e.g., respiratory-, gastro-intestinal-, and urogenital tracts) that segregate the outside world from the inside of the body are constantly exposed to environmental antigens. Such surfaces are continuously monitored for intrusion of foreign antigens by a specialized compartment of the immune system, the Mucosa-Associated Lymphoid Tissue (MALT) [1]. Bearing similar, yet distinct, structural organization as secondary lymphoid tissues (e.g., regional lymph nodes and the spleen), initiation of a B cell response in MALT preferentially leads to production of antigen-specific dimeric IgA that is transported across the epithelial layer via the polymeric immunoglobulin receptor (pIgR) into mucosal fluids and confers protection through hindering attachment of antigen(s) to epithelial cells [2]. Additionally, antigens encountered at the mucosa can also elicit IgG antibody responses that are readily detectable in plasma/serum [3,4,5]. In contrast to this natural route of infection, antigen exposure via a systemic route (e.g., intramuscular immunization) primarily elicits an IgG response. IgG can penetrate most tissues of the body, where it serves to limit antigen dissemination through direct neutralization, facilitation of phagocytosis through generation of immune complexes, opsonization, activation of complement, or by mediating directed killing of antigen-expressing cells by natural killer cells through antibody-dependent cell-mediated cytotoxicity (ADCC) [6]. Notably, IgA is also present in plasma/serum, but exists predominantly as a monomer, and possesses similar effector functions as IgG, such as antigen neutralization, complement fixation, and the ability to interact with myeloid cells via binding with FcαRI [7,8,9].

Due to alternative routes of entry and stimulatory context, mucosal and systemic antigen exposure can elicit different CD4^+^ helper T cell responses. Whereas mucosal antigen exposure can induce tolerogenic responses, such as causing induction of nasal- [10,11,12] or oral tolerance [13], systemic encounter more commonly induces Th1 or Th2 type CD4^+^ helper T cell responses that are dependent on the nature of “danger” signals associated with the antigen [14,15,16,17]. As the location of the antigen encounter (mucosal vs. systemic) and the specific cytokine milieu in the priming environment following natural infection with SARS-CoV-2 vs. COVID-19 mRNA vaccination differ fundamentally, we tested the hypothesis that the resulting B_mem_ directed against Spike (S-antigen) protein would also differ.

Depending on the type of CD4^+^ T cell help provided during the primary immune response (guided by “danger” signals present during T cell priming/polarization), naïve B cells (that express IgM) undergo instructed Ig class switching to downstream heavy chain constant regions encoding IgA, IgE, or IgG [18]. Notably, in humans, there are four IgG subclasses (IgG1/IgG2/IgG3/IgG4) that each possess distinct effector functions due to their variable engagement with FcγR expressed by cells of the innate immune system [19]. Importantly, Ig class switching is an irreversible process that leads to permanent excision of all upstream IgH gene elements from the loci [18]. Thus, while naïve B cells are uncommitted prior to antigen encounter, the resulting effector- and memory B cell (B_mem_) progeny are biased in their Ig expression/secretion; although subsequent switching to downstream Ig class/subclass encoding genes is still possible in some instances [20].

To unambiguously address our hypothesis that S-antigen-reactive B_mem_ will differ after primary SARS-CoV-2 infection vs. COVID-19 mRNA vaccination, and in order to circumvent additional complexities arising as a consequence of subsequent S-antigen exposures (e.g., booster vaccination and/or breakthrough infections), we restricted our study to individuals for whom we could verify that the induction of S-antigen-reactive B_mem_ occurred either during the first wave of the COVID-19 epidemic in subjects with PCR-verified infection or, concerning the vaccinated cohort, to individuals who were immunized shortly after the vaccine become widely available (April/May 2021), before widespread infections with SARS-CoV-2 Omicron variants occurred [21].

There are numerous publications describing serum antibodies after SARS-CoV-2 infection or COVID-19 mRNA vaccination (reviewed in [22,23]). For reasons anchored in basic B cell biology, however, serum antibodies do not accurately reflect on B_mem_ generation. This is because the emergence of the ensuing plasma cell progeny (the source of plasma/serum antibodies) and B_mem_ follow distinct instructed differentiation pathways [24] (Supplemental Figure S1). When naïve B cells first encounter their cognate antigen in secondary lymphoid tissues (e.g., draining lymph nodes), they become activated and proliferate (clonal expansion). Following interactions with follicular T helper cells, some of these activated B cells migrate into germinal centers, where they undergo further rounds of cell division. Additionally, Ig class switching and acquisition of somatic hypermutations in the variable (antigen-binding) domains of their B cell receptor (BCR) also occur. As a consequence of the latter, the daughter cells of a single B cell can express a BCR with either a higher, lower, or (in spite of somatic mutations) an unaltered affinity for the antigen. Of these mutated subclones, those that have an increased affinity for the antigen will be positively selected for differentiation into the plasma cell lineage, while subclones not meeting the high affinity criterion will instead preferentially differentiate into B_mem_ [24,25]. Whereas the affinity-based selection for plasma cell differentiation serves to improve the efficacy of the ensuing antibody response against the invading (homotypic) virus, the broadened specificity of B_mem_ arising as a consequence of sustained germinal center reactions also increases the likelihood that B_mem_ with reactivity against future antigenic variants are also generated [26]. Even if the frequency of B_mem_ with specificity for an emerging variant strain is low amongst the memory repertoire, their frequency is still increased relative to naïve B cells; i.e., they exist in a clonally expanded state, are already class-switched, and hence can generate faster anamnestic (secondary-type) antibody responses following encounter with antigenic variants [25]. Consequently, to achieve a holistic understanding of B cell-mediated immune protection and specifically the ability of a pre-immune host to recognize future antigenic variants, studies of plasma/serum antibodies alone cannot substitute for studies of B_mem_ themselves [27].

**Figure 1:**
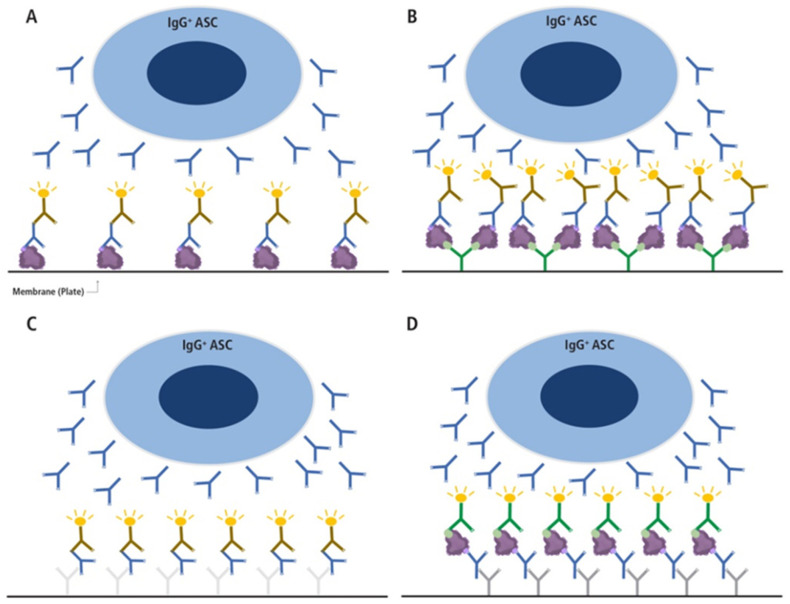
Different types of B cell ImmunoSpot assays. (**A**,**B**) Direct antigen-specific assay variants, (**C**) the pan Ig detecting test, and (**D**) the inverted antigen-specific assay. In the direct antigen-specific assay variant (**A**), the antigen itself is coated onto the membrane directly, as has been performed traditionally, whereas in variant (**B**), the antigen’s binding to the membrane is aided by high-affinity capture utilizing an affinity tag. For the latter, a His-tagged recombinant protein (depicted as a purple blob and the His-tag epitope denoted in light green) is captured onto the membrane with high affinity via the plate-bound anti-His antibody (depicted in green). In A and B, only Ig produced by antigen-specific antibody-secreting cells (ASCs) with sufficient binding affinity will be retained on the lawn of antigen bound on the membrane (the ASC-derived antibodies are depicted in blue in all panels) and are visualized by adding an anti-human Ig detection antibody (in the example shown, anti-IgG, depicted in brown). In the pan Ig detecting assay (**C**), the Ig produced by ASC is captured by an anti-species antibody coated onto the membrane (e.g., a goat-anti-human Igκ/λ, depicted in light grey), and the plate-bound human IgG is visualized using an anti-human IgG Fc-specific detection antibody (depicted in brown). In this assay variant, as with the inverted assay (**D**), secretory footprints generated by IgG^+^ ASC are captured irrespective of their antigen-specificity. In the inverted assay shown (**D**), the membrane is coated with an anti-human IgG Fc-specific capture antibody (depicted in gray) and the soluble antigen (depicted as a purple blob and the His-tag epitope denoted in light green) will only be captured by secretory footprints generated by antigen-specific IgG^+^ ASC. The membrane-bound antigen is detected in a subsequent step via a detection reagent; in the example shown, the His-tagged recombinant antigen is detected via an anti-His tag-specific detection antibody (depicted in green). This figure was reproduced with permission from Kirchenbaum, *Cells*; published by MDPI, 2024 [27].

While studies of B_mem_ are necessary to achieve a better understanding of B cell-mediated immunity, there remains a paucity of such. The reason is that such studies, unlike antibody measurements, have been challenging. Technical difficulties to overcome include the low frequency of B cells specific for any given antigen, even when the B cells exist in a clonally expanded state within all PBMC. Classic approaches like growing individual clones/hybrids are limited by their labor requirements and low throughput [28]. Single cell paired *IgH*/*IgL* sequencing is also a promising novel approach but only provides indirect data on the frequency and specificity of B_mem_ unless the antibodies are recombinantly expressed and subsequently tested for their actual binding properties as only a fraction of the B cells identified may actually be antigen specific [29]– an effort again too demanding for comprehensive immune monitoring in sizeable donor cohorts. Staining antigen-specific B cells with fluorophore-labelled antigen probes followed by flow cytometric analysis is also a promising approach; however, it is frequently hampered by the complexities of achieving specific probe staining and the detection limit for cells that occur at very low frequencies [29]. Finally, ELISPOT/FluoroSpot (collectively called ImmunoSpot^®^) in theory bypasses the limitations of the above tests. Importantly, while both antigen probe staining and ImmunoSpot^®^ rely on different modalities of detection, when both are optimized, they provide concordant data for detecting S-antigen-specific B_mem_ (see Supplemental Figure S2). As the flow cytometry-based approach, however, requires about two times more PBMC and labor investment, and is also substantially more costly, we elected here to focus on ImmunoSpot^®^. In spite of these advantages, ImmunoSpot^®^ assays have not been sufficiently developed to a point where they could be used for routine monitoring of B_mem_. The primary reason for this is that in the original implementation (refer to Figure 1A), this technique often failed to detect antigen-specific B_mem_ following their differentiation into antibody-secreting cells (ASCs) due to insufficient antigen coating of the assay membrane [30]. Realizing that many antigens, including the receptor binding domain (RBD) of SARS-CoV-2 S-antigen or nucleocapsid (NCAP), do not bind in sufficient density when coated directly onto the plates and thus cannot reveal the secretory footprints originating from individual antigen-specific B cells, we introduced an affinity coating strategy (refer to Figure 1B) that has made ImmunoSpot^®^-based detection of B_mem_ universally feasible [30]. Taking advantage of this new development, along with additional refinements in the assay procedure itself [31,32,33], in this communication, we performed an in-depth analysis of B_mem_ repertoires generated following recovery from SARS-CoV-2 infection or after prime-boost COVID-19 mRNA vaccination of previously immunologically naïve subjects.

## 2. Materials and Methods

### 2.1. Human Subjects

Peripheral blood mononuclear cell (PBMC) samples originating from three defined cohorts were characterized in this study. The first cohort, collected prior to 1 November 2019, constituted the pre-COVID-19 samples. The second cohort, collected from convalescent human subjects following verification of SARS-CoV-2 infection by polymerase chain reaction (PCR) testing, constituted the post-infection cohort. Samples from both the pre-COVID-19 and post-infection cohorts were collected at FDA-registered collection centers and were obtained from IRB-consented healthy human donors by leukapheresis and then were sold to CTL, identifying donors by code only while concealing the subjects’ identities. PBMC were cryopreserved according to previously described protocols [33]. Additionally, blood samples from a third cohort were collected internally at CTL under an Advarra-approved IRB #Pro00043178 (CTL contract laboratory study number GL20-16 entitled COVID-19 Immune Response Evaluation), and PBMC were isolated and cryopreserved according to previously described protocols [33]. All PBMC samples were stored in liquid nitrogen until testing. Details of all human donors included in this manuscript, including demographics, collection dates, and duration since SARS-CoV-2 infection or COVID-19 mRNA vaccination, are provided in Supplemental Table S1. Of note, it would have been more ideal to have an increased number of COVID-19 mRNA vaccinated samples to better balance the sample sizes of each cohort. However, such SARS-CoV-2 infection “negative” subjects’ blood needed to have been cryopreserved Fprior to the widespread of the virus, and such samples can no longer be obtained in present day. To this end, we tested all of the qualifying PBMC samples that we had access to. Similarly, in order for us to study the primary B cell response elicited following infection or vaccination with the prototype Wuhan-Hu-1 strain Spike (S-antigen) protein (denoted as WH1-S), any bleeds occurring after the emergence of SARS-CoV-2 variants would fall outside of the scope of our current manuscript, and this precluded further longitudinal sampling of the vaccination cohort to track the stability of WH1-S-specific B_mem_. As these days, individuals lacking B_mem_ reactivity for the S-antigen (along with NCAP) are very rare (according to our ongoing research efforts), the data presented in this communication were obtained using a very unique set of PBMC samples. Still, we believe the number of subjects in each of the three cohorts (*n* = 12 in the pre-COVID-19, *n* = 16 in the SARS-CoV-2 infected, and *n* = 8 in the COVID-19 mRNA vaccinated cohorts) was sufficient to identify major differences among the groups, while we acknowledge that subtle differences might go undetected. Lastly, as recent evidence surfaced that IgA^+^ B_mem_ do not effectively recirculate [34], testing nasopharyngeal swabs in parallel to blood would have been ideal and may have revealed larger differences between the infected and vaccinated cohorts. Regrettably, collection of such samples was outside the scope of IRB-approved protocols under which the samples were obtained.

### 2.2. Polyclonal B Cell Stimulation

Detailed methods of thawing, washing, and counting of PBMC have been previously described [33]. Cells were seeded into polyclonal B cell stimulation cultures within 2 h of thawing. Freshly thawed PBMC samples were resuspended in complete medium containing RPMI 1640 (Alkali Scientific, Fort Lauderdale, FL, USA) supplemented with 10% fetal bovine serum (Gemini Bioproducts, West Sacramento, CA, USA), 100 U/mL penicillin, 100 U/mL streptomycin, 2 mM L-Glutamine, 1 mM sodium pyruvate, 8 mM HEPES (all from Life Technologies, Grand Island, NY, USA), and 50 µM β-mercaptoethanol (Sigma-Aldrich, St. Louis, MO, USA). PBMC were then stimulated with Human B-Poly-S (CTL, Shaker Heights, OH, USA) containing TLR7/8 agonist R848 and recombinant human IL-2 [35] at 0.5–2 × 10^6^ cells/mL in 25 cm^2^ or 75 cm^2^ tissue culture flasks (Corning, Sigma-Aldrich) and incubated at 37 °C and 5% CO_2_ for 5 days to promote terminal differentiation of resting B cells into antibody-secreting cells (ASCs) prior to evaluation in ImmunoSpot^®^ assays.

### 2.3. Recombinant Proteins

Recombinant full-length SARS-CoV-2 Spike (S-antigen) protein representing the ancestral Wuhan-Hu-1 strain [36], denoted as WH1-S (FL), or a truncated version encoding only the receptor binding domain (RBD) [37], denoted as WH1-S (RBD), were acquired from the Center for Vaccines and Immunology (CVI) (University of Georgia (UGA), Athens, GA, USA). RBD protein representing the Omicron variant (BA.1), denoted as BA.1-S (RBD), was purchased from Creative Biomart (Shirley, NY, USA). Recombinant SARS-CoV-2 Nucleocapsid (NCAP) protein was purchased from the Wuhu Interferon Biological Products Industry Research Institute (Wuhu, China). Importantly, all recombinant proteins used in this study possessed a genegenetically encoded affinity tag.

### 2.4. B Cell ImmunoSpot^®^ Assays

#### 2.4.1. Multiplexed Antigen-Specific FluoroSpot Assays with Affinity Capture Coating

For detection of antigen-specific ASCs in multiplexed FluoroSpot assays using the affinity capture coating method [30], low autofluorescence FluoroSpot assay wells were first pre-conditioned with 70% (*v*/*v*) EtOH (15 μL/well) followed by two washes with phosphate-buffered saline (PBS) (150 μL/well) prior to coating with purified anti-His antibody at 10 µg/mL in Diluent A (provided in CTL’s affinity coating kits) overnight at 4 °C. The following day, FluoroSpot assay plates were washed once with 150 μL (PBS and then coated overnight at 4 °C with His-tag labeled recombinant WH1-S (FL) or WH1-NCAP protein at 10 µg/mL in Diluent A. Prior to use, assay plates were washed once with 150 μL PBS and then blocked with complete medium for 1 h at room temperature (RT). Immediately prior to plating of cells, assay plates were decanted, and 100 μL pre-warmed complete medium was added to each well.

PBMC were collected after 5 days of polyclonal stimulation and washed twice with PBS prior to counting using CTL’s Live/Dead Cell Counting Suite on an ImmunoSpot^®^ S6 Flex Analyzer (CTL). Cell pellets were resuspended at 5 × 10^6^ live cells/mL in complete medium and used immediately in ImmunoSpot^®^ assays. To increase the WH1-S (FL) assay’s sensitivity for detecting rare antigen-reactive ASCs, pre-COVID-19 era samples were tested in three replicate wells seeded with 5 × 10^5^ polyclonally stimulated PBMC. Alternatively, post-infection or post-vaccination samples were tested using a single two-fold serial dilution approach starting at 5 × 10^5^ live cells per well. To avoid damage to the assay membrane, PBMC were serially diluted in round-bottom 96-well tissue culture plates (Corning, Sigma-Aldrich) and then subsequently transferred into assay plates, as previously described [33]. Samples from the post-infection and post-vaccination cohorts were additionally tested for WH1-S (FL)-reactive ASCs in a two-fold dilution series starting at 5 × 10^4^ PBMC per well to improve the accuracy of frequency determinations for samples with an abundance of spot-forming units (SFUs). For WH1-NCAP ImmunosSpot^®^ assays, all samples were tested in three replicate wells seeded with 5 × 10^5^ polyclonally stimulated PBMC to improve the assay’s limit of detection. Following plating of PBMC, assay plates were incubated for 16 h at 37 °C, 5% CO_2_. Plate-bound SFUs, each representing the secretory footprint of a single ASC, were visualized using either IgA, IgE-, IgG-, and IgM-specific detection reagents or IgG1-, IgG2-, IgG3-, and IgG4-specific detection reagents, according to the manufacturer’s instructions (CTL).

#### 2.4.2. Multiplexed Pan Ig Class and Subclass Detection

To verify ASC functionality in the polyclonally stimulated PBMC samples, pan Ig class (IgA/IgE/IgG/IgM) and IgG subclass (IgG1/IgG2/IgG3/IgG4) ImmunoSpot^®^ assays were set up in parallel with the antigen-specific assays detailed above. For detecting all ASCs, irrespective of their antigen specificity, cell suspensions were serially diluted two-fold in singlets, starting at 2 × 10^5^ or 2 × 10^4^ PBMC per well in round bottom 96-well tissue culture plates and subsequently transferred into low autofluorescence FluoroSpot assay plates that were coated overnight at 4 °C (following pre-wetting with 70% (*v/v*) EtOH as described above) with anti-κ/λ capture antibody contained in the human IgA/IgE/IgG/IgM Four-Color ImmunoSpot^®^ kit or IgG1/IgG2/IgG3/IgG4 Four-Color ImmunoSpot^®^ kit (from CTL). FluoroSpot plates were then washed with PBS and blocked with complete medium as described above. Following plating of PBMC, assay plates were incubated for 16 h at 37 °C and 5% CO_2_. Plate-bound SFUs, each representing the secretory footprint of a single ASC irrespective of antigen specificity, were subsequently visualized using either IgA, IgE-, IgG-, and IgM-specific detection reagents or IgG1-, IgG2-, IgG3-, and IgG4-specific detection reagents, according to the manufacturer’s instructions (CTL).

#### 2.4.3. Single-Color Inverted FluoroSpot Assays for Detection of SARS-CoV-2 S-Reactive IgG^+^ ASC

To further restrict the reactivity of S-antigen-reactive ASCs to the receptor binding domain (RBD), polyclonally stimulated PBMC samples from the post-infection and post-vaccination cohorts were also tested in IgG-specific inverted ImmunoSpot^®^ assays using His affinity-tagged WH1-S (FL) or RBD proteins representing the WH1 or Omicron variant BA.1 strains as detection probes. In brief, low autofluorescence FluoroSpot assay plates were coated overnight at 4 °C (following pre-wetting with 70% (*v*/*v*) EtOH) with anti-human IgG Fc capture antibody at 15 µg/mL in Diluent A (provided in CTL’s inverted ImmunoSpot^®^ kits). FluoroSpot plates were then washed with PBS and blocked with complete medium as described above. Polyclonally stimulated PBMC samples from the post-infection and post-vaccination cohorts were then plated in eight replicate wells at donor-specific inputs (referred to as the “Goldilocks cell input”) previously determined to yield ~50 WH1-S (FL)-reactive IgG^+^ SFUs in assays utilizing the affinity capture coating methodology. Following plating of PBMC, assay plates were incubated for 16 h at 37 °C, 5% CO_2_. After washing, His-tagged WH1-S (FL) at 2 µg/mL, WH1-S (RBD) at 100 ng/mL, or BA.1-S (RBD) at 100 ng/mL in Diluent B (provided in CTL’s inverted ImmunoSpot^®^ kits) was added into the designated wells, respectively, and incubated for 2 h at RT. After decanting and washing, anti-His detection antibody conjugated with Alexa Fluor^®^ 488 (provided in CTL’s His inverted ImmunoSpot^®^ kit) was added to the designated wells and incubated for 1 h at RT to visualize individual S-antigen-reactive SFUs.

#### 2.4.4. FluoroSpot Image Acquisition and SFU Counting

FluoroSpot plates were air-dried prior to scanning on an ImmunoSpot^®^ Ultimate S6 Analyzer using the Fluoro-X suite of ImmunoSpot^®^ software (Version 7.0.28) (CTL). Quantification of SFUs in assay wells was performed using ImmunoSpot^®^ Studio.SC software (Version 1.7.28.0) and B cell IntelliCount™ algorithms [38] or the Basic Count mode. Individual well images were quality controlled to remove artifacts and improve accuracy of counts as needed. Only SFU counts within the linear titration range of the ImmunoSpot^®^ assay, or SFU counts from the highest cell input tested, were considered for frequency calculations and were subsequently used to extrapolate SFU counts to a fixed input of 10^6^ PBMC. As ImmunoSpot^®^ Multi-color B cell kits, analyzers, and software proprietary to CTL were used in this study, we refer to the collective methodology as ImmunoSpot^®^.

### 2.5. Statistical Methods

Statistically significant differences in the frequency of WH1-S (FL)- and WH1-NCAP-specific ASCs were determined using unpaired *t*-tests (GraphPad Prism 10 Version 10.4.0, San Diego, CA, USA) and are denoted in the corresponding figure legends. To determine the relative frequency of WH1-S (FL)-reactive IgG^+^ ASCs that recognized epitopes in the RBD region of the WH1 or BA.1 variant strains, the cumulative SFU count from replicate wells probed with the WH1-S (FL) protein was designated as the 100% value. The cumulative SFU counts from replicate wells probed with either the WH1-S (RBD) or BA.1-S (RBD) proteins were then divided by this SFU value (corresponding to 100% of the WH1-S (FL)-reactive response at the donor-specific PBMC input) and are expressed as a percentage.

## 3. Results and Discussion

### 3.1. Establishing the Frequency of SARS-CoV-2 Spike (S)- and Nucleocapsid (NCAP)-Reactive IgG^+^ B_mem_ in Defined PBMC Cohorts

Having developed an affinity coating strategy (Figure 1B) that enabled detection of secretory footprints from SARS-CoV-2 S-antigen- or NCAP-reactive IgG^+^ B_mem_ [30], here, we sought to more stringently define the characteristics of these antigen-specific cells. To this end, and accounting for possible chance cross-reactivity with common cold coronaviruses (CCC) that were in circulation prior to the emergence of SARS-CoV-2 [39], ImmunoSpot^®^ assays enabling visualization of secretory footprints generated by IgG-secreting B cells with reactivity to a full-length S-antigen representing the ancestral Wuhan-Hu-1 strain, denoted as WH1-S (FL), or WH1- NCAP protein were performed using polyclonally stimulated PBMC originating from three defined cohorts (pre-COVID-19, post-infection, or post-vaccination; detailed below). Importantly, because, in previous studies, we observed that frequencies of WH1-S-reactive IgG^+^ B_mem_ in convalescent subjects were present over a broad frequency range [40], PBMC from the post-infection or post-vaccination cohorts were tested for reactivity against the WH1-S (FL) protein using a single well serial dilution strategy starting at 5 × 10^5^ PBMC per well [32]. Alternatively, a fixed cell input of 5 × 10^5^ PBMC in three replicate wells was used for testing the pre-COVID-19 cohort for WH1-S (FL)-reactive ASC to improve the limit of detection for rare ASCs. Lastly, since frequencies of WH1-NCAP-reactive ASCs tended to be lower than those specific for the S-antigen, PBMC samples from all cohorts were tested at a fixed input of 5 × 10^5^ PBMC in three replicate wells.

The first PBMC cohort detailed in this communication, constituting pre-COVID-19 era blood draws (cryopreserved before 1 November 2019) might exhibit chance cross-reactivity with third-party antigens, such as those corresponding to antigens expressed by CCC, but importantly, would not have been exposed to SARS-CoV-2 itself due to their date of collection prior to the first confirmed SARS-CoV-2 infection in the United States [41]. The second PBMC cohort originated from convalescent donors who recovered from PCR-verified SARS-CoV-2 infections in the early months of 2020 at the onset of the COVID-19 epidemic and before vaccines became widely available. These individuals would be expected to have developed B_mem_ to both WH1-S (FL) and WH1-NCAP proteins. The third PBMC cohort consisted of those who were prime-boost immunized with COVID-19 mRNA vaccine (encoding the WH1-S protein) soon after it became available in April/May 2021 and before widespread SARS-CoV-2 infections with the Omicron variant strains occurred [21]. In these subjects, we expected to detect WH1-S (FL) but not WH1-NCAP-reactive IgG^+^ B_mem_ (unless some of these individuals had subclinical SARS-CoV-2 infection, in addition, for which we controlled through testing the polyclonally stimulated PBMC samples for IgG^+^ ASC reactivity against the WH1-NCAP protein).

**Figure 2:**
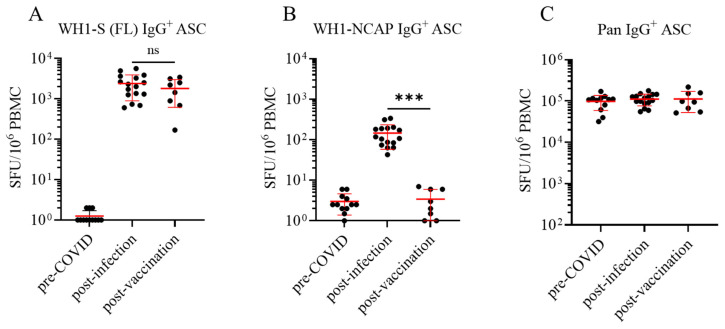
Frequency of WH1-FL S- and NCAP-specific IgG^+^ ASC in defined cohorts of cryopreserved PBMC. Peripheral blood mononuclear cells (PBMCs) collected from SARS-CoV-2-infected (*n* = 16, denoted as post-infection cohort) and COVID-19 mRNA-vaccinated (*n* = 8, denoted as post-vaccination cohort) or pre-COVID-19 era controls (*n* = 12) were subjected to in vitro polyclonal stimulation with Human B-Poly-S (R848+rIL-2) to convert resting B cells into antibody-secreting cells (ASCs) for subsequent evaluation in multiplexed B cell FluoroSpot assays (refer to Section 2.2 and Section 2.4). (**A**) Frequencies of IgG^+^ ASCs specific for a full-length SARS-CoV-2 Spike protein representing the Wuhan-Hu-1 strain, denoted as WH1-S (FL), were calculated using spot-forming unit (SFU) counts occurring in the linear range and extrapolated to SFU per 10^6^ PBMC. Each data point denotes an individual donor in the respective cohorts, and the mean ± SD for each cohort is shown in red. Donors in the pre-COVID-19 cohort lacking detectable WH1-S (FL)-reactive IgG^+^ ASCs were assigned a value of 1 SFU per 10^6^ PBMC for graphing purposes. Statistical significance (*** *p* < 0.001) of the difference between pre-COVID-19 era and post-infection or post-vaccinated PBMC samples was determined using an unpaired *t*-test; note, the frequencies of WH1-S (FL)-specific IgG^+^ ASCs in the infected and vaccinated cohorts were not significantly different (*p* = 0.34). (**B**) Frequencies of IgG^+^ ASCs specific for the SARS-CoV-2 WH1 Nucleocapsid protein, denoted as WH1-NCAP, are expressed as SFU per 10^6^ PBMC. Statistical significance (*** *p* < 0.001) of the difference between infected and vaccinated donor cohorts was determined using an unpaired *t*-test. (**C**) Frequencies of pan (total) IgG^+^ ASCs, detected irrespective of antigen specificity (refer to Figure 1C), are expressed as SFU per 10^6^ PBMC.

For IgG measurements, such ImmunoSpot^®^ assays revealed exquisite specificity (Figure 2A,B): None of the pre-COVID-19 era PBMC harbored appreciable WH1-S (FL)- and WH1-NCAP-reactive IgG-producing B_mem_—thus, no false positives were seen. These data also suggest that prior CCC infection(s), had they occurred in our pre-COVID-19 era donors, failed to elicit a sizeable population of IgG^+^ B_mem_ with cross-reactivity for the WH1-S (FL) antigen. In contrast, all PBMC samples from the post-infection and post-vaccination cohorts contained WH1-S (FL)-reactive IgG^+^ B_mem_, and notably, the frequencies of such cells were similar in the two cohorts at the time of testing (3–7 months after induction of the B cell responses). As expected, NCAP-reactive IgG^+^ B_mem_ were detected in all of the previously infected donors, albeit at much lower frequencies than were detected for WH1-S (FL). However, NCAP-reactive IgG^+^ B_mem_ were absent in tests utilizing PBMC from the pre-COVID-19 or post-vaccination cohorts. These data support the notion that the post-vaccination cohort had neither been infected with SARS-CoV-2 prior to their vaccination nor during the elapsed time between their vaccination and the blood collections. Therefore, as was the intent of this study, the primary induction of WH1-S (FL)-reactive IgG^+^ B_mem_ was what was indeed being monitored in the post-infection and post-vaccination cohorts.

Notably, measurement of B_mem_ secreting antibody of undefined specificity in pan IgG assays (Figure 2C, refer to Figure 1C for the assay principle) performed in parallel confirmed that pre-COVID-19 era PBMC samples were indeed functional and hence capable of successful differentiation into IgG^+^ ASCs following polyclonal stimulation. Therefore, the failure to detect WH1-S (FL)- or WH1-NCAP-reactive IgG^+^ B_mem_ in pre-COVID-19 era samples was not a consequence of their impaired IgG^+^ ASC activity. Moreover, pre-COVID-19 era samples exhibited similar frequencies of B_mem_-derived IgG^+^ ASCs with reactivity for alternative antigens representing seasonal influenza or Epstein–Barr virus [40]. Due to the considerable inter-individual variation in the frequency of pan IgG^+^ ASCs following polyclonal stimulation of PBMC, we also present the frequency of WH1-S (FL)-reactive and WH1-NCAP-reactive IgG^+^ B_mem_-derived ASCs amongst all IgG^+^ ASCs (Supplementary Figure S3).

### 3.2. Defining Ig Classes of B_mem_ Elicited by Natural Infection vs. Vaccination

**Figure 3:**
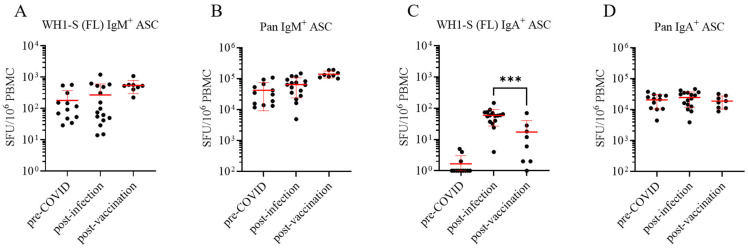
Assessment of WH1-S (FL)-reactive IgM^+^ and IgA^+^ ASCs in defined cohorts of cryopreserved PBMC. Peripheral blood mononuclear cells (PBMCs) collected from SARS-CoV-2-infected (*n* = 16), COVID-19 mRNA-vaccinated (*n* = 8), or pre-COVID-19 era controls (*n* = 12) were evaluated for reactivity against the WH1-S (FL) protein in multiplexed B cell FluoroSpot assays as described in Figure 2 (refer to Section 2.4 for additional details). Frequencies of IgM^+^ (panel (**A**)) and IgA^+^ (panel (**C**)) ASCs reactive to WH1-S (FL) are expressed as SFU per 10^6^ PBMC. Donors lacking detectable WH1-S (FL)-reactive IgA^+^ ASCs were assigned a value of 1 SFU per 10^6^ PBMC for graphing purposes. Frequencies of pan (total) IgM^+^ (panel (**B**)) and IgA^+^ (panel (**D**)) ASCs, irrespective of antigen specificity, are expressed per 10^6^ PBMC. In all panels, mean ± SD for each cohort is denoted in red. Statistical significance (*** *p* < 0.001) of the difference between infected and vaccinated donor cohorts was determined using an unpaired *t*-test Note: Results for IgE are not shown since they were negative, including the lack of pan IgE^+^ ASC detection.

Immunoglobulins (Igs) occur in the classes IgM, IgD (expressed by naïve B cells, primarily), as well as IgG, IgA, and IgE, which are expressed on the surface of resting B cells constituting their BCR, or are secreted following their differentiation into an ASC. Surface expression or secretion of a class-switched Ig (IgG, IgA, or IgE) is an indicator that a B cell has previously participated in a classical T cell-dependent immune response. As the different classes of antibodies play fundamentally different roles in host defense, we compared Ig class utilization of the WH1-S (FL)-reactive B_mem_ generated following SARS-CoV-2 infection or COVID-19 mRNA vaccination, along with PBMC samples from the pre-COVID-19 era cohort as controls. Four-color multiplexed ImunoSpot^®^ assays were performed to enable simultaneous detection of IgM-, IgG-, IgA-, or IgE-secreting cells (note that these assays offer similar sensitivity as single-color ELISPOT tests [32,33]). Using such four-color assays, we characterized the WH1-S (FL)-reactive B cell repertoires in the three cohorts. In contrast to the clear-cut IgG results shown in Figure 2, the frequency of WH1-S (FL)-reactive IgM^+^ ASCs was similar in all three cohorts (Figure 3A); i.e., such cells were detected in the pre-COVID-19 era donors as well, a finding suggesting that these IgM^+^ ASCs are not derived from antigen-experienced B_mem_ but instead likely reflect the presence of low-affinity, broadly cross-reactive naïve B cells that differentiated into ASCs following polyclonal stimulation. The presence of IgM^+^ WH1-S (FL) reactive ASCs in pre-COVID-19 donors, as well as the occurrence of pan IgM^+^ ASCs in similar numbers in all three cohorts (Figure 3B), provided further evidence for the unimpaired functionality of the pre-COVID-19 PBMC samples.

WH1-S (FL)-reactive IgA^+^ ASCs (whose mere presence, like for IgG and unlike for IgM, implies that such cells have undergone antigen-driven T cell-dependent Ig class switching) were readily detected in 15 of 16 samples from the post-infection cohort but only in 4 of 8 samples from the post-vaccination cohort and at significantly lower frequencies (Figure 3C). The similar frequencies of pan IgA^+^ ASCs between the three donor cohorts (Figure 3D) further confirmed that the functionality of the PBMC samples cannot account for the observed differences in WH1-S (FL)-reactive IgA^+^ B_mem_ frequencies. Overall, frequencies of WH1-S (FL)-reactive IgA^+^ B_mem_-derived ASCs were considerably lower than the frequency of WH1-S (FL)-reactive IgG^+^ B_mem_-derived ASCs in both the convalescent and vaccinated cohorts. These data are also in line with the notion that SARS-CoV-2 infection of the upper respiratory tract mucosa and lung elicited a more robust induction of IgA^+^ B_mem_ circulating in peripheral blood compared to intramuscular injection of the COVID-19 mRNA vaccine. We contend that analogous B cell ImmunoSpot^®^ assays as described herein would be ideally-suited to comprehensively address the question of whether SARS-CoV-2 infection or prime-boost COVID-19 mRNA vaccination were equally capable of increasing the frequency of WH1-S (FL)-reactive IgA^+^ or IgG^+^ B_mem_ present in nasopharyngal swabs, or in draining lymph node, bronchus-associated lymphoid tissue (BALT), or lung biopsy samples, but this could not be tested here.

Lastly, due to the inclusion of IgE-specific detection reagents in the multiplexed B cell ImmunoSpot^®^ assays, we can also report the failure to detect WH1-S (FL)-reactive IgE^+^ ASC in any of the samples. Related to this, pan IgE^+^ ASCs, irrespective of antigen specificity, were not detected either, consistent with the notion that circulating IgE^+^ B_mem_ may not exist (or possibly, the generation of IgE^+^ ASCs requires de novo class switching [20]). Consistent with this notion, we also observed that polyclonal stimulation of B cells in vitro under conditions that mimic T cell help (in the presence of agonistic anti-CD40 antibody plus IL-4 and IL-21) induced IgE^+^ ASCs that were clearly detectable at single-cell resolution using an equivalent ImmunoSpot^®^ detection system [33]. In aggregate, these observations indicate that had pan IgE^+^ ASCs been present in our polyclonally stimulated PBMC samples at frequencies exceeding the lower detection limit of the assay as performed, i.e., 1 in 2 × 10^5^ PBMC, they would have been detected.

### 3.3. Dissecting IgG Subclass Utilization of WH1-S (FL)-Reactive B_mem_ in Infected or Vaccinated Subjects

IgG antibodies can be segregated into four subclasses, whose expression also depends on Ig class switching. Importantly, Ig expression is restricted through the process of allelelic exclusion; i.e., an individual B cell can express only one Ig class/subclass [42]. The four IgG subclasses trigger distinct engagement of innate immune reactions [19]. Namely, IgG1 and IgG3 excel in complement fixation and the induction of Fc-receptor-induced phagocytosis and ADCC. In contrast, IgG2 exhibits reduced affinity for FcγRs, particularly FcγRIIIb, and is less effective at triggering ADCC. However, under conditions of high antigen density, IgG2 is still capable of activating the complement cascade. Lastly, IgG4 possesses a reduced affinity for FcγR and does not efficiently trigger classical extra-neutralizing effector functions. Instead, IgG4 may possess anti-inflammatory properties through its ability to compete with other IgG subclasses for antigen binding. Notably, IgG4 can undergo Fab-arm exchange, which renders it functionally monovalent [43]. Therefore, assessing the IgG subclass(es) expressed by WH1-S (FL)-reactive B_mem_ that were generated following SARS-CoV-2 infection vs. COVID-19 mRNA vaccination would unveil critical information regarding the type of protective immunity elicited by each.

**Figure 4:**
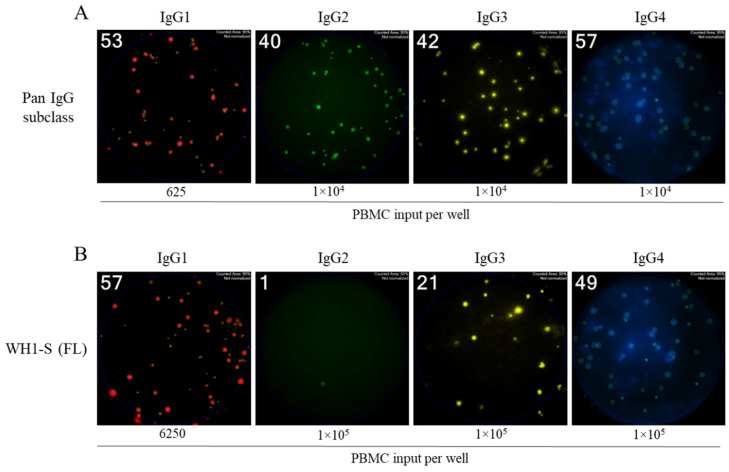
Four-color ImmunoSpot^®^ assays enable parallel detection of IgG subclass usage. Representative well images from four-color multiplexed FluoroSpot assays enabling detection of “pan” antibody-secreting cells (ASCs) irrespective of their antigen specificity (panel (**A**)) or those secreting WH1-S (FL)-specific IgG (panel (**B**)). Cell inputs are specified below the corresponding images, which were contrast-enhanced to aid visualization. Depicted well images have diameter of ~6mm.

**Figure 5:**
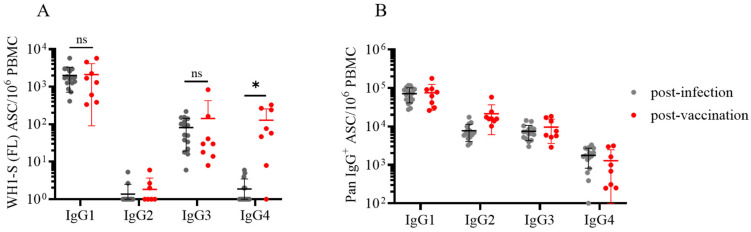
Evaluation of WH1-S (FL)-specific ASC IgG subclass usage in SARS-CoV-2-infected or COVID-19 mRNA-vaccinated donors. Peripheral blood mononuclear cells (PBMCs) collected from SARS-CoV-2-infected (*n* = 16) or COVID-19 mRNA-vaccinated (*n* = 8) donors were evaluated for reactivity against the WH1-S (FL) protein in multiplexed B cell FluoroSpot assays as described in Figure 2 (refer to Section 2.4 for additional details). (**A**) Frequency of WH1-S (FL)-specific ASCs producing IgG1, IgG2, IgG3, or IgG4 is expressed as SFU per 10^6^ PBMC. Frequencies of WH1-S (FL)-specific IgG1^+^ or IgG3^+^ ASCs in the infected or vaccinated donor cohorts were not significantly different, whereas the frequency of WH1-S (FL)-specific IgG4^+^ ASCs was significantly increased (* *p* < 0.05) in the vaccinated donor cohort by unpaired *t*-tests. (**B**) Frequencies of pan (total) ASCs, irrespective of antigen specificity, producing IgG1, IgG2, IgG3, or IgG4 are expressed as SFU per 10^6^ PBMC. In both panels (A and B), mean ± SD for each cohort is denoted in red. Notably, the apparent absence of WH1-S (FL)-specific IgG2^+^ ASCs was not attributable to impaired detection since pan IgG2^+^ ASCs were present in all samples (see also Figure 4A).

To this end, we performed multiplexed ImmunoSpot^®^ assays unambiguously detecting all four IgG subclasses: Examples of representative wells are shown in Figure 4 and Supplementary Figure S4. As noted above, we had previously established that such four-color assays facilitated detection of secretory footprints with equal sensitivity to that of single-color ELISPOT measurements [32,33]. Consequently, parallel assessment of IgG subclass usage was enabled without increasing the amount of cell material required. Both the post-infection and post-vaccination cohorts displayed clearly elevated frequencies of WH1-S (FL)-reactive IgG1^+^ ASCs (Figure 5A) at similar frequencies. Also, both donor cohorts possessed detectable WH1-S (FL)-reactive IgG3^+^ ASCs, albeit at much lower frequencies than IgG1^+^ ASCs. However, WH1-S (FL)-reactive IgG2^+^ ASCs were essentially undetectable in both the post-infection and post-vaccination samples despite verification of IgG2^+^ ASC activity when measured irrespective of their antigen specificity (Figure 5B). Notably, the lack of detectable WH1-S (FL)-reactive IgG2^+^ ASCs is not consistent with prior observations [44,45] that reported S-antigen binding IgG2 in serum samples collected from COVID-19 mRNA vaccinated subjects. While a different IgG2-specific detection reagent was used in these studies than our own, our inability to detect WH1-S-reactive IgG2^+^ ASC cannot be attributed to the usage of a lower-affinity detection reagent based on our own internal testing. Furthermore, using equivalent assay conditions, we detected a low frequency of WH1-S (FL)-reactive IgG2^+^ ASCs in a PBMC sample obtained from a previously vaccinated subject acutely following a PCR-verified SARS-CoV-2 infection that occurred early in 2022 during the initial spread of the Omicron variant [21] (see Supplemental Figure S5). Lastly, while the frequencies of pan IgG4^+^ ASCs were similar in both cohorts (Figure 5B), WH1-S (FL)-reactive IgG4^+^ ASCs were significantly increased only in the post-vaccination cohort (Figure 5A).

Recently, increased attention has been focused on the emergence of IgG4 antibodies following repeated COVID-19 mRNA vaccinations [46]. While addressing the consequences of chronic antigen exposure is beyond the scope of this communication, we believe the observation that the post-vaccination cohort possessed an increased frequency of WH1-S (FL)-reactive IgG4^+^ B_mem_-derived ASCs after only the initial prime-boost vaccination regimen is noteworthy because this observation was made at the memory B cell level whereas earlier reports detected this phenomenon in serum samples [44,45,47].

In ongoing work, we also identified several individuals exhibiting an increased frequency of WH1-S (FL)-reactive IgG4^+^ ASCs after receiving multiple COVID-19 mRNA vaccinations (Kirchenbaum, work in progress). Shifting of the IgG subclass usage directed against the SARS-CoV-2 S-antigen is likely to have biological consequences since IgG4 binds FcγR with reduced affinity compared to IgG1 or IgG3, and consequently, IgG4 is less efficient at mediating FcγR-dependent effector mechanisms [47,48]. In support of this notion, elevated titers of S-antigen binding IgG4 were associated with an increased risk for breakthrough infection [49].

We include Supplementary Figure S6 to better illustrate—by showing data on a linear scale—how variable the frequencies of WH1-S (FL)-reactive ASCs producing different Ig classes or IgG subclasses are; four representative donors in the post-infection or post-vaccination study cohorts are shown. The data also reiterate the requirement to perform serial dilutions when performing frequency measurements for the individual Ig classes and subclasses.

### 3.4. Characterization of RBD-Reactive B_mem_ Induced in Infected or Vaccinated Subjects

While antibodies targeting any exposed region of a viral antigen can contribute to host defense [50,51], antibodies that are endowed with neutralizing activity are of particular significance [52]. In the most simplified scenario, a virus infects a permissive target cell by first adhering to a dedicated target receptor. For SARS-CoV-2, the homotrimeric Spike glycoprotein (also commonly referred to as the S-antigen) expressed on infectious virions mediates binding to target cells via an interaction with the host receptor, angiotensin-converting enzyme 2 (ACE2) [53]. More specifically, the SARS-CoV-2 S-antigen possesses a stretch of amino acids in the S1 subunit that is designated the “receptor binding domain” (RBD) and which binds to ACE2 with nanomolar affinity [54]. Antibodies targeting the SARS-CoV-2 RBD can therefore disrupt the association of S-antigen with ACE2 and correlate with neutralizing activity [55,56]. When comparing the B_mem_ elicited in donors following infection or vaccination, it therefore seemed critical to further restrict the investigation of B_mem_ to those recognizing the RBD region of the WH1-S protein.

**Figure 6:**
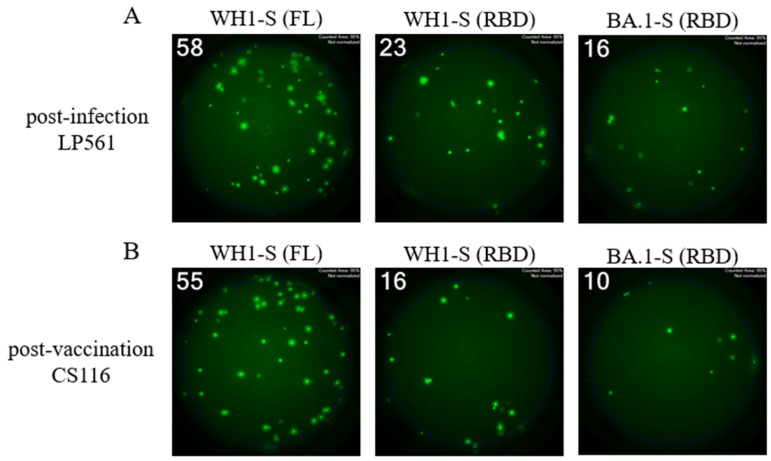
Single-color inverted FluoroSpot enables detection of RBD-reactive IgG^+^ ASCs. Representative well images from inverted assays (refer to Figure 1D) in which polyclonally stimulated PBMCs were seeded at a “Goldilocks” cell input previously determined to yield ~50 SFUs in a direct assay using affinity capture coated WH1-S (FL) protein. Results obtained using the specified probes for a post-infection donor (panel (**A**)) or a post-vaccination donor (panel (**B**)) are shown. Refer to Section 2.4.3 for additional assay details. Depicted well images have diameter of ~6mm.

Due to the WH1-S (RBD) construct being a shorter polypeptide relative to WH1-S (FL), as well as it being a monomer vs. the trimerized WH1-S (FL) probe, we found WH1-S (RBD) less suitable for direct coating. Even when coated using an affinity capture strategy (illustrated in Figure 1B and with which the WH1-S (FL) and WH1-NCAP data described so far were generated), we failed to detect pristine secretory footprints after direct coating with WH1-S (RBD). We therefore leveraged an alternative strategy referred to as the “inverted assay” (illustrated in Figure 1D) for measuring the frequencies of WH1-S (RBD)-reactive B_mem_-derived ASCs. In this ImmunoSpot^®^ assay variant, the ASCs are seeded onto membranes coated with capture antibodies that are specific for an Ig class of interest. In our case, since IgG^+^ ASCs were the most prevalent WH1-S-reactive B_mem_ population, assay membranes were coated with an anti-human IgG Fc-specific capture reagent. Thus, similar to pan Ig-detecting assays (illustrated in Figure 1C), all IgG^+^ ASCs are capable of generating a secretory footprint irrespective of their antigen specificity. Following removal of the cells, the antigen of interest is then added and serves as the detection probe, being selectively captured by only the secretory footprints originating from antigen-specific IgG^+^ ASCs. Since IgG^+^ ASCs with irrelevant specificity greatly outnumber the antigen-specific ones following polyclonal stimulation of PBMCs, such inverted assays are ideally performed using lower cell inputs than direct assays to avoid local saturation of the capture antibodies’ capacity. As shown in Figure 6, inverted ImmunoSpot^®^ assays performed utilizing either WH1-S (FL) or WH1-S (RBD) as detection probes (performed under optimal test conditions—see below) enabled visualization of pristine secretory footprints. However, using higher cell inputs, we observed suboptimal secretory footprint formation, resulting as a consequence of interference between the high density of neighboring ASCs, and/or elevated membrane staining from the recapture of WH1-S (RBD)-reactive antibody distally from the source ASCs.

**Figure 7:**
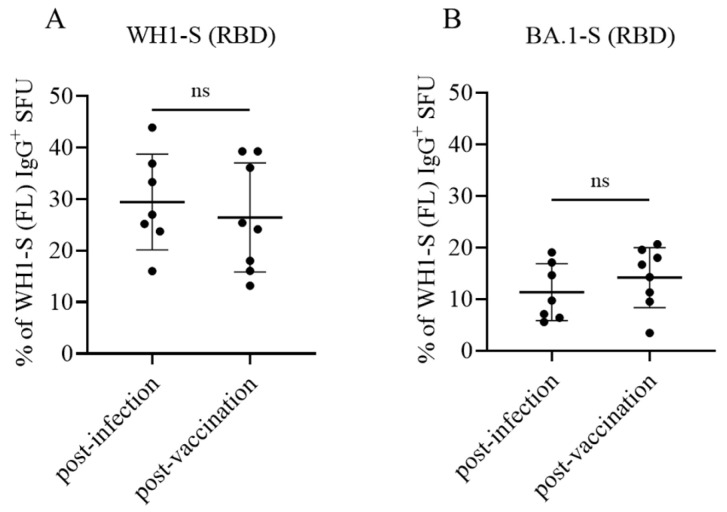
B_mem_ recognition of the WH1 (homotypic) or BA.1 (heterotypic) receptor binding domain (RBD). PBMC collected from SARS-CoV-2-infected (*n* = 7) or COVID-19 mRNA-vaccinated (*n* = 8) donors were seeded into IgG-specific inverted ImmunoSpot^®^ assays following polyclonal stimulation at donor-specific “Goldilocks” cell inputs previously determined to yield ~50 SFUs in a direct assay using affinity capture coated WH1-S (FL) protein (refer to Section 2.4.3 for additional details). Donor PBMC samples were seeded in replicate wells, and the cumulative number of WH1-S (FL)-reactive SFUs detected was denoted as 100% of the donor’s response. In parallel, replicate wells seeded at the same donor-specific “Goldilocks” cell input were detected using WH1-S (RBD) or BA.1-S (RBD) protein as the detection probe (see Figure 6). Percentages of WH1-S (FL)-reactive SFUs with inferred reactivity for epitopes in the WH1-S (RBD) (panel (**A**)) or BA.1-S (RBD) (panel (**B**)) are denoted, respectively. Inferred frequency of WH1-S (FL)-reactive IgG^+^ SFUs recognizing the homotypic (WH1) or heterotypic (BA.1) RBD probes were not significantly different between the post-infection or post-vaccination cohorts by two-tailed unpaired *t*-tests (*p* = 0.57 for WH1 and *p* = 0.35 for BA.1, respectively).

Similar to the direct assays described previously, inverted assays can also be performed using a serial dilution approach to define the frequency of antigen-reactive ASCs in a test sample. When we performed WH1-S (RBD) assays in parallel with those evaluating ASC reactivity against WH1-S (FL) (in this case, WH1-S (FL) was coated using affinity capture) during the initial assessment of donor samples, we obtained a low-resolution approximation of the relative frequency of WH1-S (FL)-reactive IgG^+^ ASCs that recognized epitopes within the RBD. However, having established in such assays the optimal number of PBMCs to be plated per well for each donor to obtain a “Goldilocks cell input” (~50 SFUs), in a follow-up experiment, we next sought to more precisely determine the relative frequency of WH1-S (FL)-reactive IgG^+^ ASCs that recognized the RBD. To this end, eight replicate wells were seeded with the donors’ pre-determined Goldilocks cell input, and WH1-S-reactive IgG^+^ SFUs were subsequently revealed using either the WH1-S (FL) or WH1-S (RBD) probes. Due to the increased number of replicate wells seeded with an equivalent number of PBMC, we were able to calculate the percentage of WH1-S (FL)-reactive IgG^+^ SFUs that were specific for the WH1-S (RBD) with higher resolution using this approach. Such systematic comparisons of donors from the post-infection or post-vaccination cohorts showed that in either case, ~30% the WH1-S (FL)-reactive B_mem_ repertoire targeted epitopes within the WH1-S RBD. Notably, we did not observe a significant difference between the post-infection and post-vaccination donor cohorts (Figure 7A).

Collectively, these data (namely, similar frequencies of WH1-S (FL)-reactive IgG^+^ B_mem_ recognizing epitopes in the RBD) suggest that the neutralizing potential of secondary recall responses in case of (re)-infection with the homotypic WH1 strain would be similar in both cohorts. This notion is critical, as several studies have shown that the concentrations and neutralizing capacity of plasma/serum antibodies declined after infection or the initial prime-boost COVID-19 vaccination regimen [57,58,59,60,61].

### 3.5. Comparing Omicron Cross-Reactive B_mem_ in WH1 Vaccinated vs. WH1 Infected Individuals

New variants of a virus arise as a consequence of their ability to evade pre-existing neutralizing antibody activity elicited by the previously circulating strain(s). Next to enabling affinity maturation of antibodies directed against the homotype, somatic hypermutation also serves to increase the likelihood that clonally expanded and class-switched B_mem_ capable of recognizing future variants are also generated [26]. If such cross-reactive cells are already present within the B_mem_ repertoire, upon subsequent infection with a heterotypic virus, they will enable faster, secondary-type recall antibody responses. We therefore tested to what extent the B_mem_ repertoires induced following infection or vaccination with WH1-S would cross-react with the RBD of an Omicron variant (BA.1) that emerged after these blood samples were collected [21]. Having already established for each test subject the frequency of WH1-S (RBD)-reactive B_mem_ relative to the frequency of WH1-S (FL)-reactive B_mem_ through performing inverted assays using serially diluted PBMC, as an additional component to our higher resolution follow-up experiment (see above), we also included additional replicate wells to reveal the number of BA.1-S (RBD) probe-reactive IgG^+^ ASCs. In line with the large number of immune-evading mutations acquired within the RBD region of the BA.1 Omicron variant [62,63], we observed that both post-infection and post-vaccination samples exhibited a similarly low frequency (ranging between 5 and 20%) of WH1-S-reactive IgG^+^ B_mem_-derived ASCs capable of recognizing the BA.1-S (RBD) probe (Figure 7B). These data demonstrate that a comparable subset of the WH1-S-reactive IgG^+^ B_mem_ generated following either infection or vaccination would be capable of recognizing epitopes maintained within the RBD region of BA.1 and therefore would have the potential to contribute towards an anamnestic host defense reaction following a breakthrough infection [64,65].

## 4. Conclusions

Enabled by new developments in the field of B cell ImmunoSpot^®^ analysis, we provide here a comprehensive analysis of WH1-S-antigen-reactive B_mem_ elicited in the simplest immune scenario, in previously naïve subjects following their first infection with the original “prototype” or the original COVID-19 mRNA vaccine encoding the same WH1-S-antigen. Perhaps surprisingly from the standpoint of basic immunology expectations, the B_mem_ repertoires generated in these two cohorts were found to be quite similar, although the biological significance of the subtle differences seen in IgA^+^ and IgG4^+^ B_mem_ populations is not clear and warrants further investigation. This study was restricted to sample cohorts for which defined exposure histories were available. This “first of its kind” systematic characterization of B cell memory provides basic insights into B_mem_ development under defined conditions, contrasting natural infection with vaccination. At the same time, it was intended to demonstrate the feasibility of such ImmunoSpot^®^-based immune monitoring for studies of more complex immunologic scenarios, such as following several booster injections and/or recurrent infections with newly circulating variant strains. This report should catalyze future efforts aimed at achieving a more comprehensive assessment of antigen-specific B_mem_ in large donor cohorts (which, mostly for technical reasons, have not been hitherto undertaken) due to their key contributions to acquired immunity.

## Data Availability

The data generated in this study will be made available by the authors, without undue reservation, to any qualified researcher.

## Supplementary Materials

The following supporting information can be downloaded at: https://www.mdpi.com/article/10.3390/vaccines13090944/s1, Figure S1: Affinity determines the distinct differentiation pathways of plasma vs. memory B cells; Figure S2: Concordance between flow cytometric probe staining and ImmunoSpot^®^ assays for detection of class-switched S-antigen-specific memory B cells; Figure S3: Frequency of WH1-S (FL)- and NCAP-specific IgG+ ASC in defined cohorts of cryopreserved PBMC; Figure S4: Specificity of Four-color ImmunoSpot^®^ IgG subclass-specific detection systems; Figure S5: Detection of low frequency WH1-S (FL)-specific IgG2+ ASC in a Four-color ImmunoSpot^®^ assay; Figure S6: Detection of WH1-S (FL)-reactive ASCs producing different Ig classes/IgG subclasses shown on a linear scale; Table S1: Donor demographics.
