# Supplementary material for: SARS-CoV-2 Infection or COVID-19 mRNA Vaccination Elicits Partially Different Spike-Reactive Memory B Cell Responses in Naïve Individuals
Source: Vaccines (Basel). 2025 Sep 3;13(9):944. doi: 10.3390/vaccines13090944 (PMC12474496; doi:10.3390/vaccines13090944)
Supplement: Supplementary file 1 [file vaccines-13-00944-s001.zip › vaccines-3805963-supplementary.pdf]

Supplementary Materials:

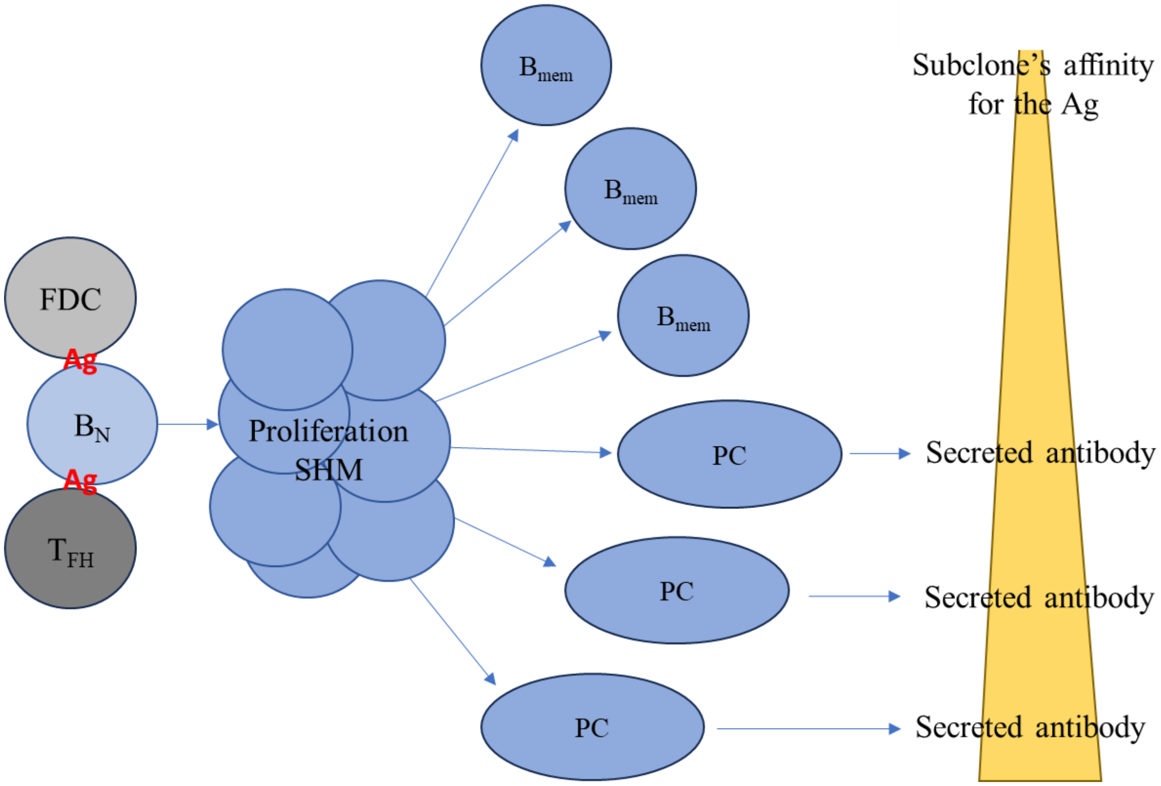

Supplemental Figure S1. Affinity determines the distinct differentiation pathways of plasma vs. memory B cells. A naïve B cell (B_N) encounters the homotypic antigen (Ag) (in a draining lymph node or spleen) for which its antigen receptor (BCR) has significant affinity (e.g., > K_D^{-5}) and becomes activated following cognate interactions with follicular T helper cells (T_{FH}) and follicular dendritic cells (FDCs). The B cell then enters a germinal center (GC), where it clonally expands, undergoes Ig class switching, and acquires somatic hypermutation (SHM) of its BCR. As SHM is a random process, the progeny of the original B_N cell express BCRs with a broad spectrum of affinities for the homotypic antigen. Daughter cells (subclones) expressing high-affinity BCRs are retained in the GC, and after multiple rounds of cell division, further SHM, and positive selection, they eventually differentiate into plasma cells (PCs). Progeny with BCRs of lower affinity for the homotypic antigen exit the GC as memory B cells (B_{mem}). As a consequence, B_{mem} with lower affinity for the homotypic antigen may be endowed with an increased affinity for a heterotypic antigen variant that may be encountered in the future.

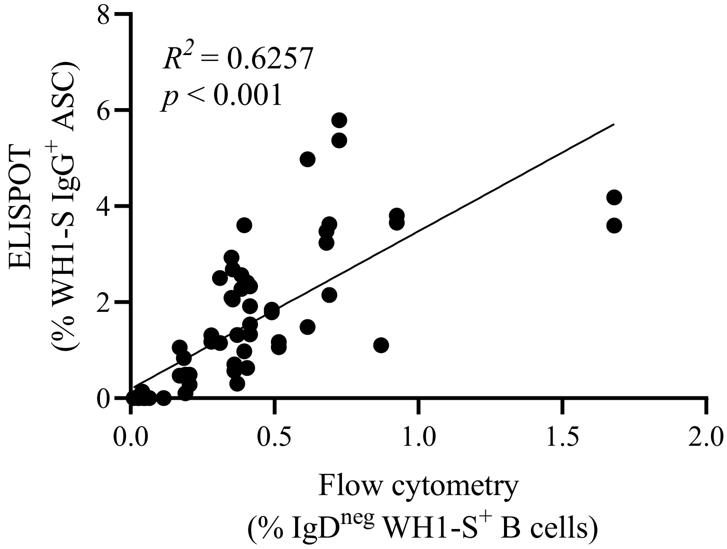

Supplemental Figure S2. Concordance between flow cytometric probe staining and ImmunoSpot® assays for detection of class-switched S-antigen-specific memory B cells. Cryopreserved peripheral blood mononuclear cells (PBMC) (n = 36) were thawed and 10^7 cells allocated for surface staining with PE- and APC-conjugated WH1-S probes, along with additional surface markers, to facilitate identification of S-antigen probe binding IgD^{neg} B cells via flow cytometry (G. Stylianou, manuscript in preparation). Alternatively, 5×10^6 PBMC were subjected to in vitro polyclonal stimulation with Human B-Poly-S (R848+rIL-2) to convert resting B cells into antibody-secreting cells (ASCs) and were subsequently evaluated for IgG$^+$ ASC reactivity against the WH1-S (FL) protein in single-color ELISPOT assays. R^2 and p-value from Pearson correlation analysis is denoted in the figure inset. Notably, both technical approaches for detection of S-antigen-specific memory B cells provided concordant data using common starting material for the respective assays.

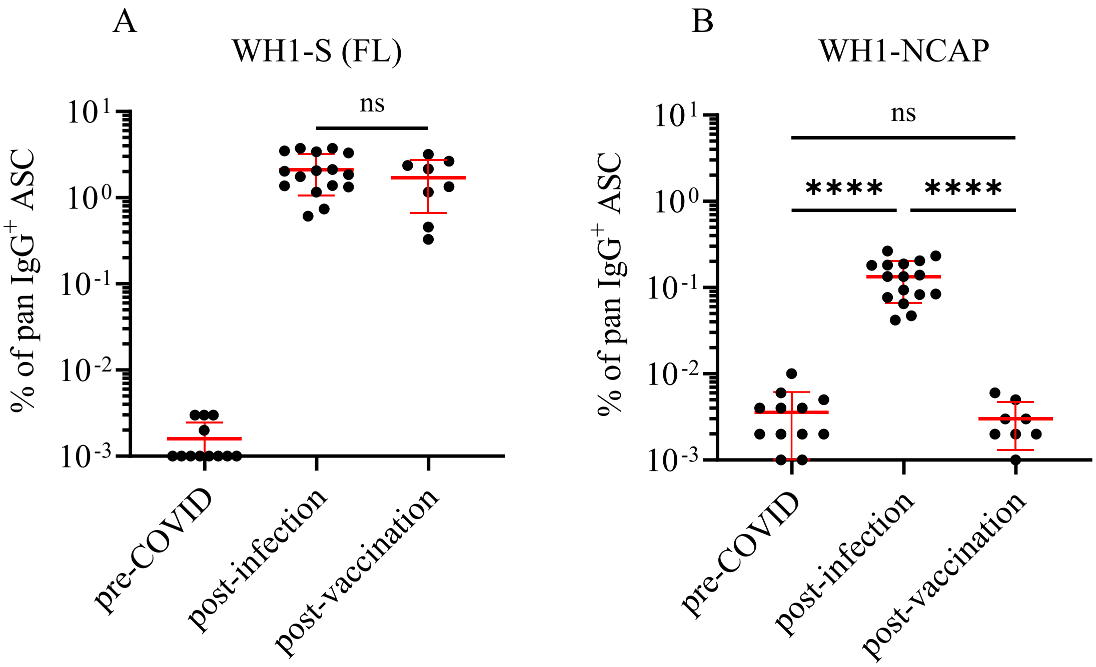

Supplemental Figure S3. Frequency of WH1-S (FL)- and NCAP-specific IgG$^+$ ASC in defined cohorts of cryopreserved PBMC. A and B) Peripheral blood mononuclear cells (PBMC) collected from SARS-CoV-2 infected subjects (n = 16), COVID-19 mRNA vaccinated (n = 8) or pre-COVID era controls (n = 12) were evaluated for reactivity against the WH1-S (FL) and WH1-NCAP proteins in multiplexed B cell FluoroSpot assays as described in Section 2.4. A) Frequency of IgG$^+$ ASC with reactivity for WH1-S (FL) are expressed as a percentage of the pan (total) IgG$^+$ ASC response detected for each donor. Notably, the frequencies of WH1-S (FL)-reactive IgG$^+$ ASCs in the post-infection and post-vaccination cohorts were not significantly different (p = 0.36) by unpaired t-test. B) Frequency of IgG$^+$ ASCs with reactivity for the SARS-CoV-2 WH1 Nucleocapsid (NCAP) protein expressed as a percentage of the pan (total) IgG$^+$ ASC response. Statistical significance (*** $p < 0.001$) between post-infection and pre-COVID era or post-vaccination cohorts was determined using an unpaired t-test; however, the frequencies of WH1-NCAP-reactive IgG$^+$ ASCs in the pre-COVID and post-vaccination cohorts were not significantly different (p = 0.58). In both panels (A and B), mean ± SD for each cohort is denoted in red.

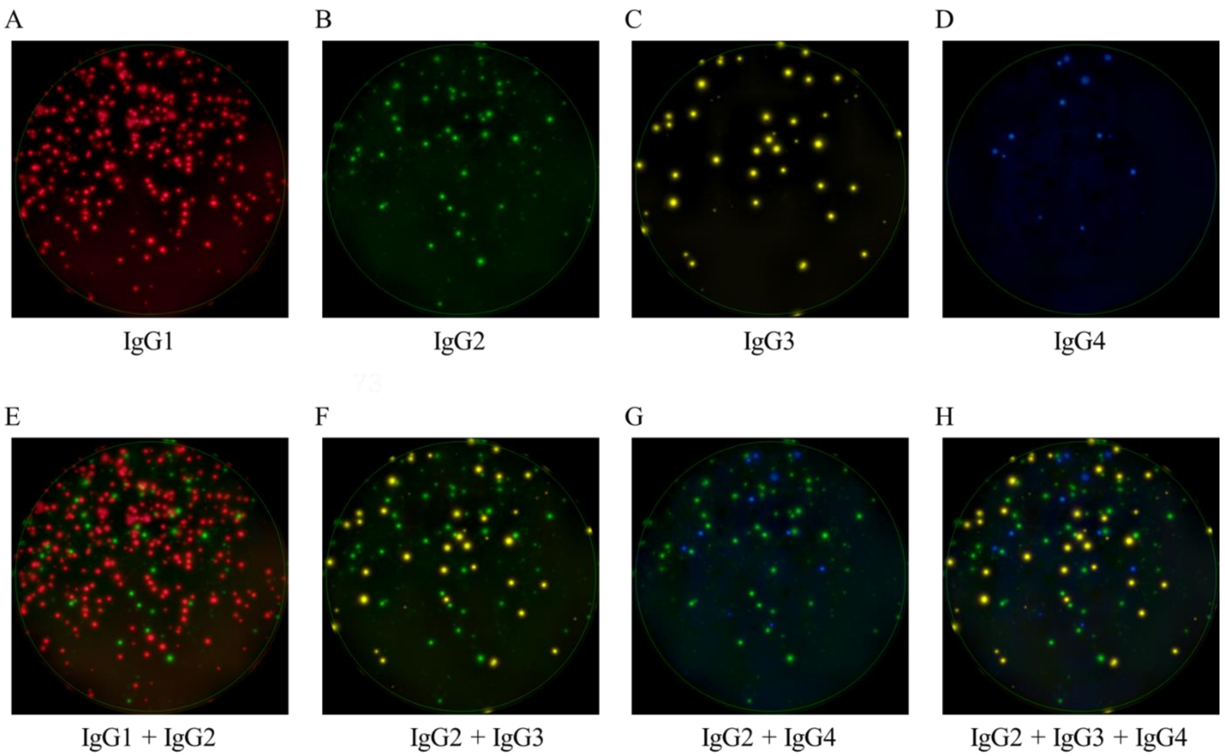

Supplemental Figure S4. Specificity of Four-color ImmunoSpot® IgG subclass-specific detection systems. A-D) Representative images of the individual IgG subclass detection planes from the same assay well seeded with ~5 × 10^3 PBMC following in vitro polyclonal stimulation with Human B-Poly-S (R848+rIL-2) to convert resting B cells into antibody-secreting cells (ASC). Note: secretory footprints were generated irrespective of the individual ASCs' antigen specificity (refer to Figure 1C for the assay principle). E-H) Virtual overlay images combining two or three fluorescent detection planes were generated using the Fluor-X suite of ImmunoSpot® software to demonstrate the exclusivity of individual spot-forming units (SFU) detected using the respective IgG subclass-specific detection systems. Consistent with the increased frequency of IgG1-expressing B cells relative to B cells expressing the other IgG subclasses, the abundance of IgG1$^+$ SFU following polyclonal stimulation of PBMC is significantly higher relative to the other IgG subclasses [32]. Depicted well images have diameter of ~6mm and were contrast-enhanced to aid visualization.

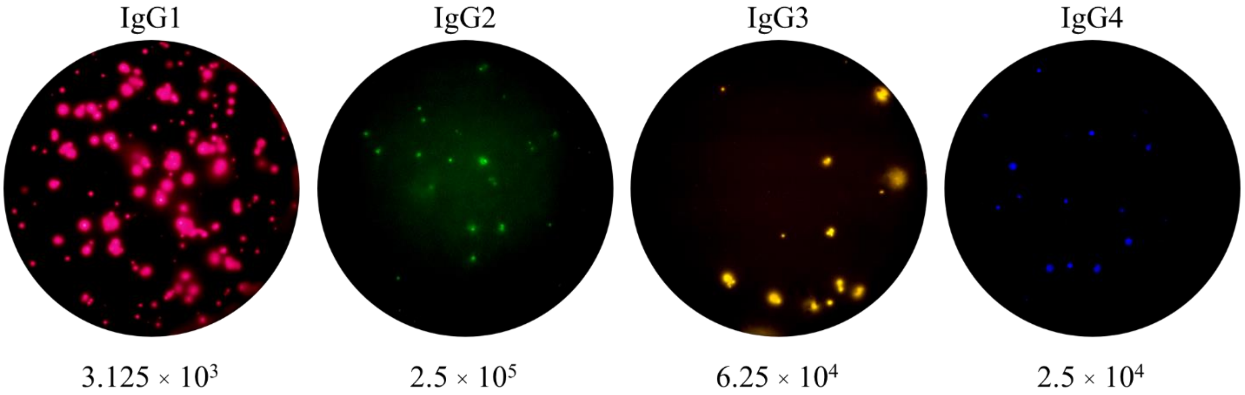

Supplemental Figure S5. Detection of low frequency WH1-S (FL)-specific IgG2⁺ ASC in a Four-color ImmunoSpot® assay. Peripheral blood mononuclear cells (PBMC) collected acutely following SARS-CoV-2 infection (in early 2022) from a previously COVID-19 mRNA vaccinated subject (CS276) were subjected to in vitro polyclonal stimulation with Human B-Poly-S (R848+rIL-2) to convert resting B cells into antibody-secreting cells (ASCs) and subsequently evaluated for reactivity against the WH1-S (FL) protein in a multiplexed B cell FluoroSpot assay (refer to Section 2.4 for additional details). Cell inputs are specified below the corresponding images. Depicted well images have diameter of ~6mm and were contrast-enhanced to aid visualization.

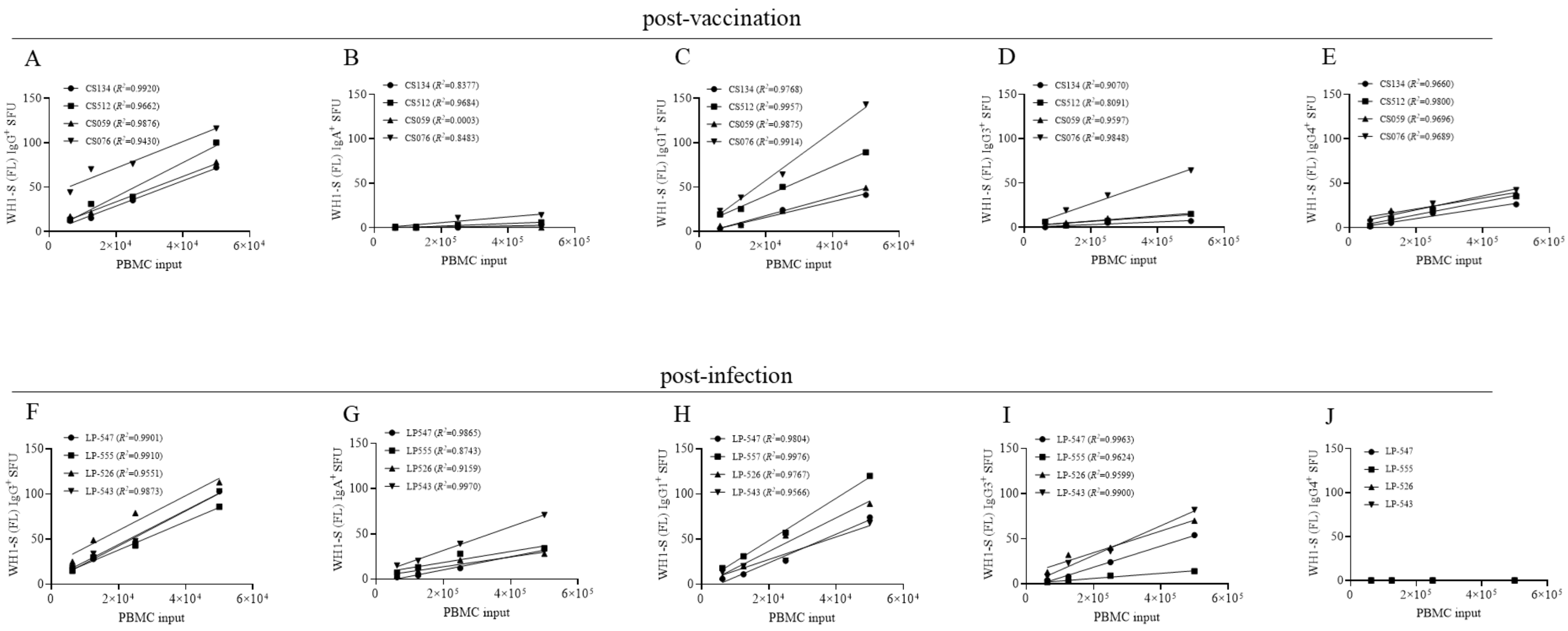

Supplemental Figure S6. Detection of WH1-S (FL)-reactive ASCs producing different Ig classes/IgG subclasses shown on a linear scale. Peripheral blood mononuclear cells (PBMC) collected from COVID-19 mRNA vaccinated individuals (denoted as post-vaccination, panels A-E depicting the same 4 donors) or SARS-CoV-2 infected (denoted as post-infection, panels F-J depicting the same 4 donors) were evaluated for reactivity against WH1-S (FL) protein in multiplexed B cell FluoroSpot assays (refer to Section 2.4.1 for additional details). Spot forming unit (SFU) counts occurring in the linear range, or for the three highest cell inputs tested, for the different Ig classes/IgG subclass are shown for each donor. Linear regression analysis was performed and the corresponding trend lines and R^2 values denoting goodness of fit for the individual data points in the dilution series are indicated in the corresponding insets of each panel.
